# Supplementary material for: A novel approach to measure mitochondrial respiration in frozen biological samples
Source: EMBO J. 2020 May 20;39(13):e104073. doi: 10.15252/embj.2019104073 (PMC7327496; doi:10.15252/embj.2019104073)
Supplement: Supplementary file 1 — Appendix [file EMBJ-39-e104073-s001.pdf]

A NOVEL APPROACH TO MEASURE MITOCHONDRIAL RESPIRATION IN FROZEN BIOLOGICAL SAMPLES

Appendix.

Appendix Figure S1-S6 and Supplemental Figure Legends

Appendix Figure S1. Mitochondria from frozen tissue are uncoupled and measure maximal OCR capacity (related to main Figure 1)

Appendix Figure S2. Spectrophotometrical Complex I activity in frozen liver mitochondria and homogenates (related to main Figure 3)

Appendix Figure S3. Frozen homogenates need cytochrome c to allow maximal respiration (related to main Figure 4)

Appendix Figure S4. RIFS validation in white adipose tissue (related to main Figure 5)

Appendix Figure S5. Validation of mitochondrial mass measurements using MTDR (related to main Figure 6)

Appendix Figure S6. RIFS optimization and validation in zebra fish muscle homogenate and human cells (related to main Figure 7)

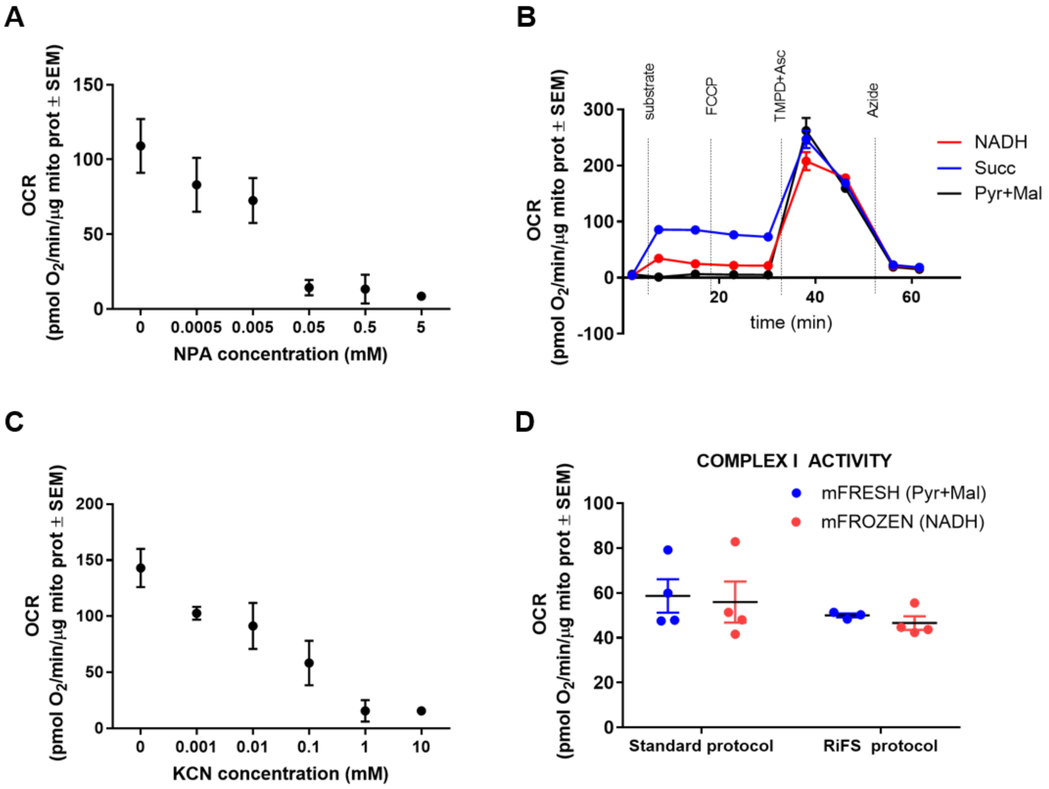

Appendix Figure S1. Mitochondria from frozen tissue are uncoupled and measure maximal OCR capacity (related to main Figure 1)

(A) Succinate + rotenone dependent respiration in frozen liver mitochondria in the presence of the Complex 2-specific inhibitor 3-nitropropionic acid (NPA) at the indicated concentrations. (B) Representative seahorse profile of frozen liver mitochondria dependent by the different substrates showing FCCP insensitivity. (C) TMPD + ascorbate dependent respiration in frozen liver mitochondria in the presence of the complex IV-specific inhibitor potassium cyanide (KCN) at the indicated concentrations. (D) Maximal complex I dependent respiration comparing the standard and RIFS seahorse protocols in fresh (mFRESH, pyruvate + malate) and frozen (mFROZEN, NADH) mitochondria. Biological replicates, n=4. Note the similar OCR of the two Complex I substrates. Every biological replicate represents the average of 4 technical replicates. Data are the mean ± SEM.

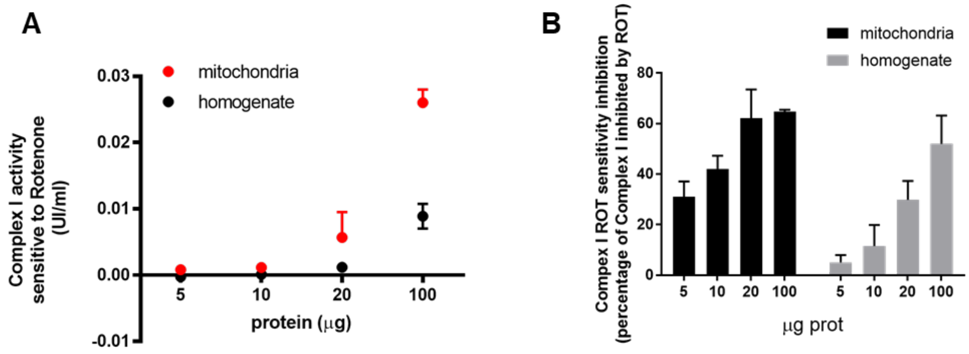

Appendix Figure S2. Spectrophotometrical complex I activity in frozen liver mitochondria and homogenates (related to main Figure 3)

(A) Rotenone sensitive complex I activity measured spectrophotometrically in frozen liver mitochondria and homogenates. (B) Percentage of NADH oxidation that is inhibited by rotenone in frozen liver mitochondria and homogenates. Biological replicates: n=4. Every biological replicate represents the average of 3 technical replicates. Data are the mean ± SEM.

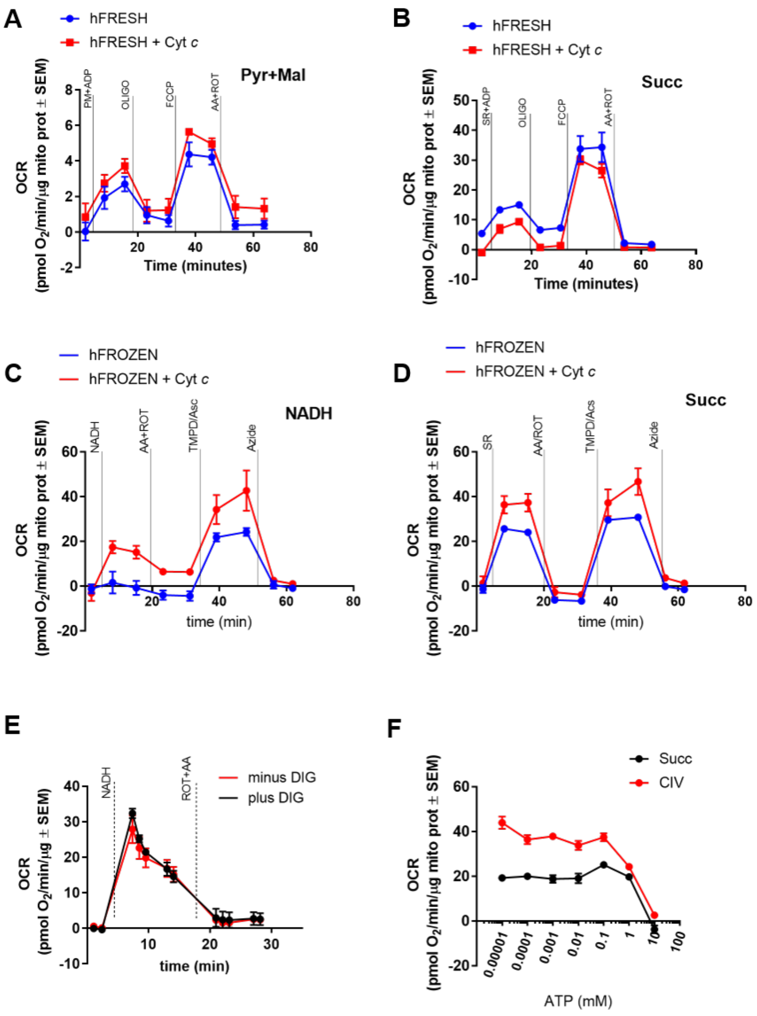

Appendix Figure S3. Frozen homogenates need cytochrome *c* to allow maximal respiration (related to main Figure 4)

Representative seahorse profile of fresh liver homogenate dependent by pyruvate + malate (A) or succinate + rotenone (B) in the presence or absence of cytochrome *c* in the assay medium. Representative seahorse profile of frozen liver homogenate dependent by NADH (C) or succinate + rotenone (D) in the presence or absence of cytochrome *c* in the assay medium. (E) NADH dependent respiration of frozen homogenate in the presence of the mild detergent digitonin. (F) Succinate + rotenone and TMPD + ascorbate dependent respiration in frozen liver mitochondria in the presence of ATP at the indicated concentrations. Biological replicates, n=4. Every biological replicate represents the average of 4 technical replicates. Data are the mean ± SEM.

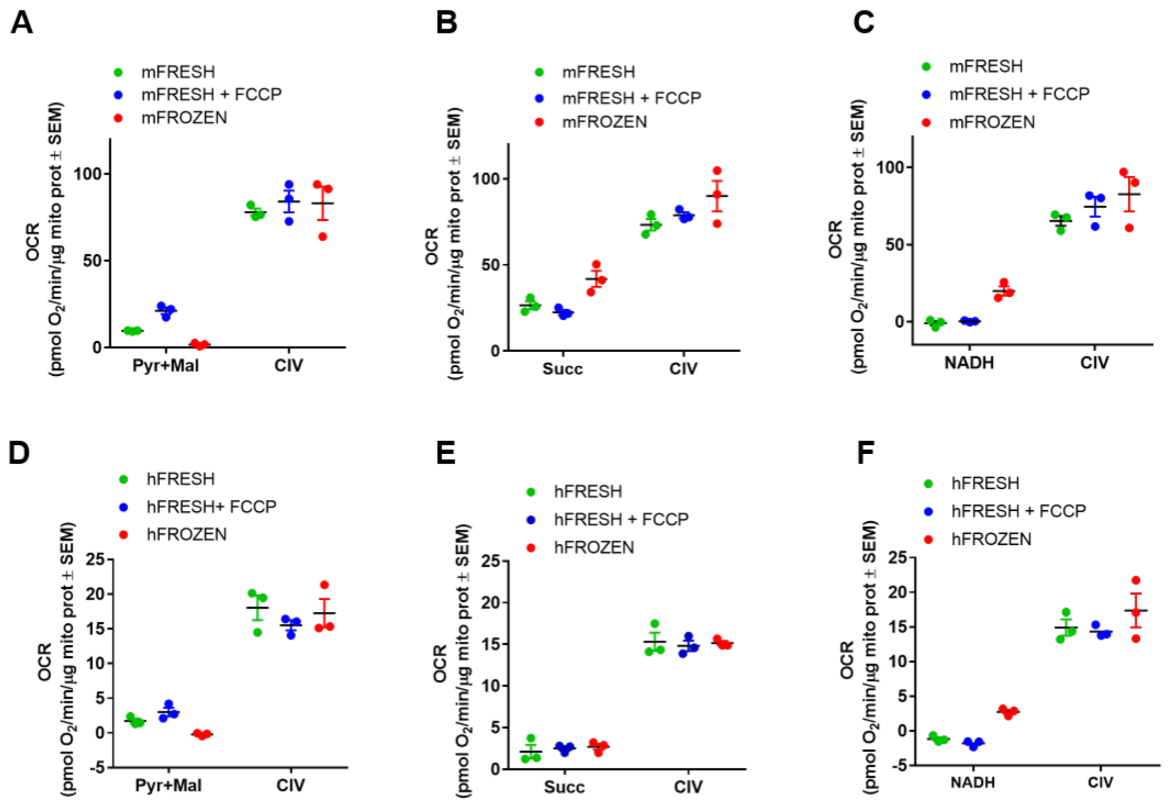

Appendix Figure S4. RIFS validation in white adipose tissue (related to main Figure 5)

(A-C) RIFS respirometry quantification in WAT mitochondria isolated from fresh (mFRESH) and frozen (mFROZEN) tissue. (D-F) RIFS respirometry quantification in WAT homogenates obtained from fresh (hFRESH) and frozen (hFROZEN) tissue. Biological replicates: n=3. Every biological replicate represents the average of 4 technical replicates. Data are the mean ± SEM.

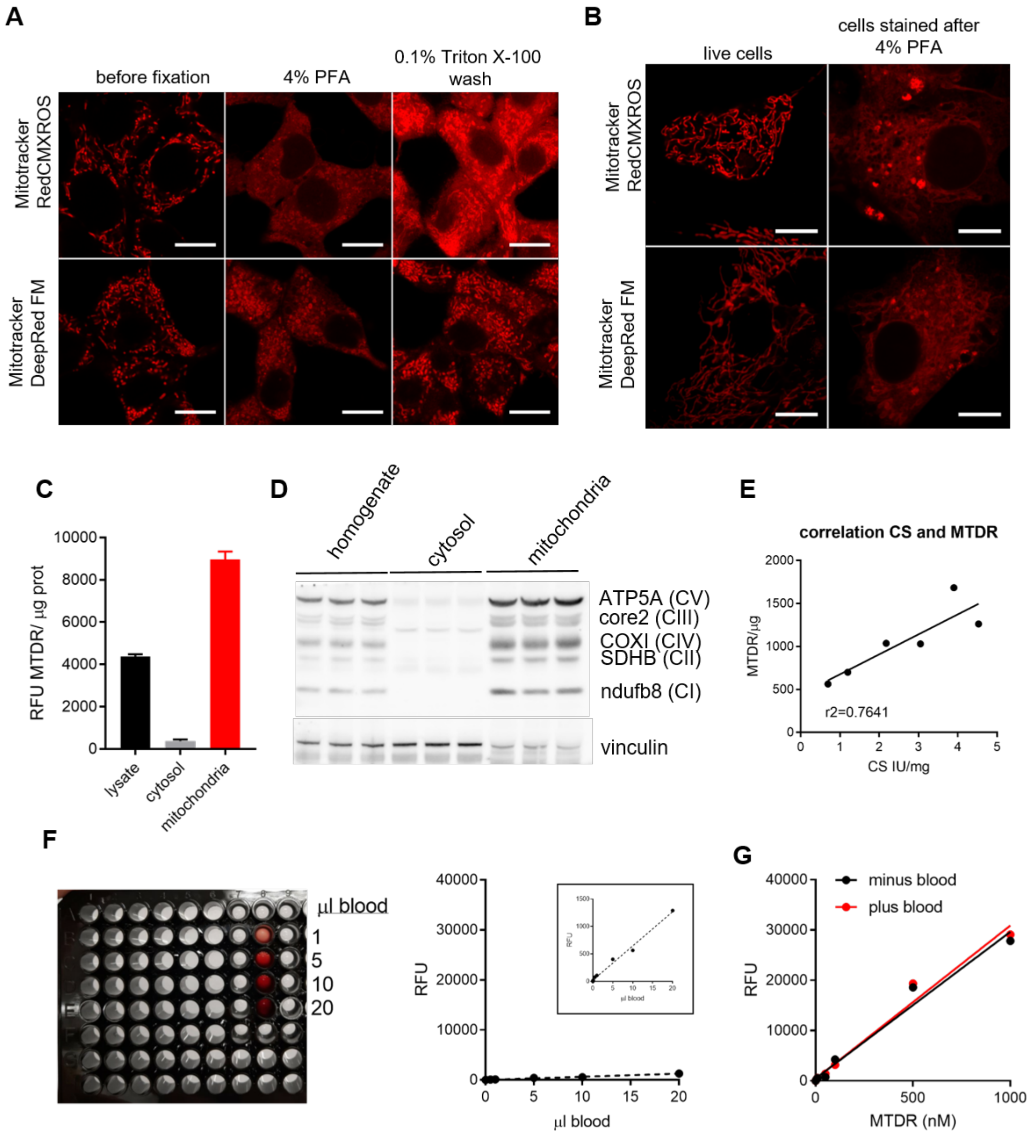

Appendix Figure S5. Validation of mitochondrial mass measurements using MTDR (related to main Figure 6)

(A) Ins1 live cells were stained with MTR or MTDR and imaged before (left panel), after fixation with 4%PFA (middle panel) and fixation plus permeabilization with Triton X (right panel). (B) Hepatic cell line Hep2G was stained with MTR or MTDR live and after fixation with PFA 4%. Scale bars represent 10 µm. MTDR fluorescence (C) and western blot (D) in total liver lysate, cytosol and mitochondrial fractions. E) Correlation between citrate synthase (CS) activity and

MTDR fluorescence in the range of 2-30 μg of lysate protein. F) Different volumes of blood plated in a 96 well plate (left panel) were used to read background noise with the same excitation/emission settings that are used to record MTDR fluorescence (right panel). The insert in the right panel demonstrates the presence of low-level, linear, concentration dependent fluorescence background. G) Fluorescence intensities of increasing concentrations of MTDR were measured using a plate reader with/without the addition of 1 μL blood solution to assess the contribution of blood background to overall MTDR fluorescence.

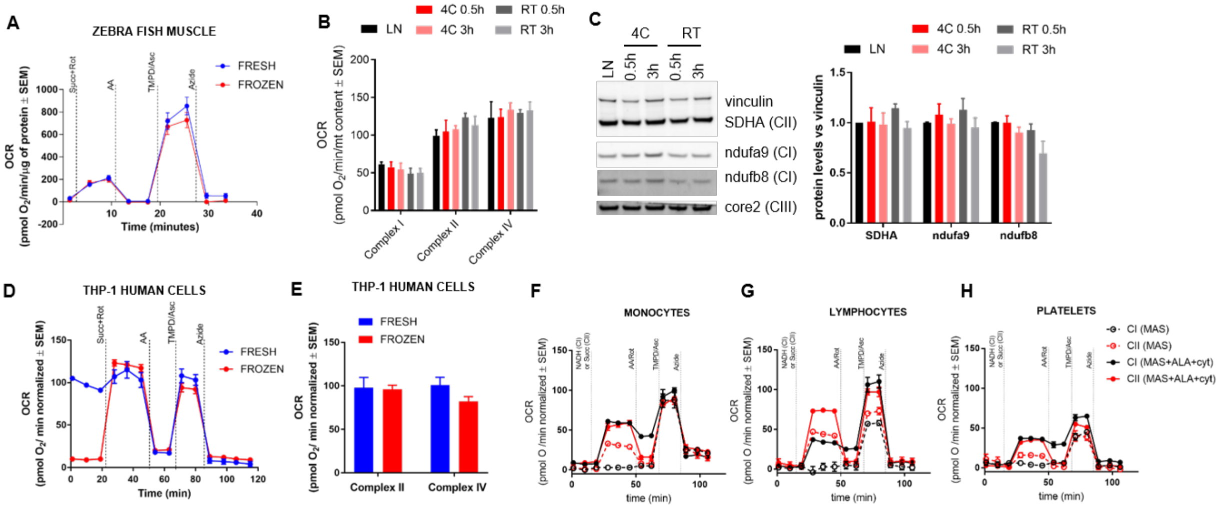

Appendix Figure S6. RIFS optimization and validation in zebra fish muscle homogenate and human cells (related to main Figure 7)

A) Representative succinate + rotenone seahorse profile using RIFS respirometry protocol in zebrafish fresh (blue) and frozen (red) muscle homogenates. B) Complex I, II and IV respiratory capacity in mouse liver lysates preserved in the indicated conditions before freezing (LN, liquid nitrogen; RT, room temperature). C) Western blot and quantification of mouse liver lysates in the indicated conditions. D) Traces of OCR by THP-1 fresh (blue) and frozen (red) for complex-II and –IV with the optimal conditions in MAS for fresh cells or MAS+ Cyt *c* +ALA 2.5 µg/ml for frozen cells, and E) the quantification of the complexes II and IV in the THP-1 fresh (blue) and frozen (red). Traces of OCR for monocytes (F), lymphocytes (G), and platelets (H) cryopreserved for the evaluation of Complex I (black) and Complex II (red) in combination with Complex IV in MAS (open circles and dashed line) versus MAS supplemented with Cyt *c* and ALA buffer (solid circle and solid line). Data are the mean ± SEM, n=5-6 per group.
