# Supplementary material for: A novel approach to measure mitochondrial respiration in frozen biological samples
Source: EMBO J. 2020 May 20;39(13):e104073. doi: 10.15252/embj.2019104073 (PMC7327496; doi:10.15252/embj.2019104073)
Supplement: Supplementary file 2 — Source Data for Appendix [file EMBJ-39-e104073-s006.zip › Appendix_SF6_blots.pdf]

Supplementary Figure 6C

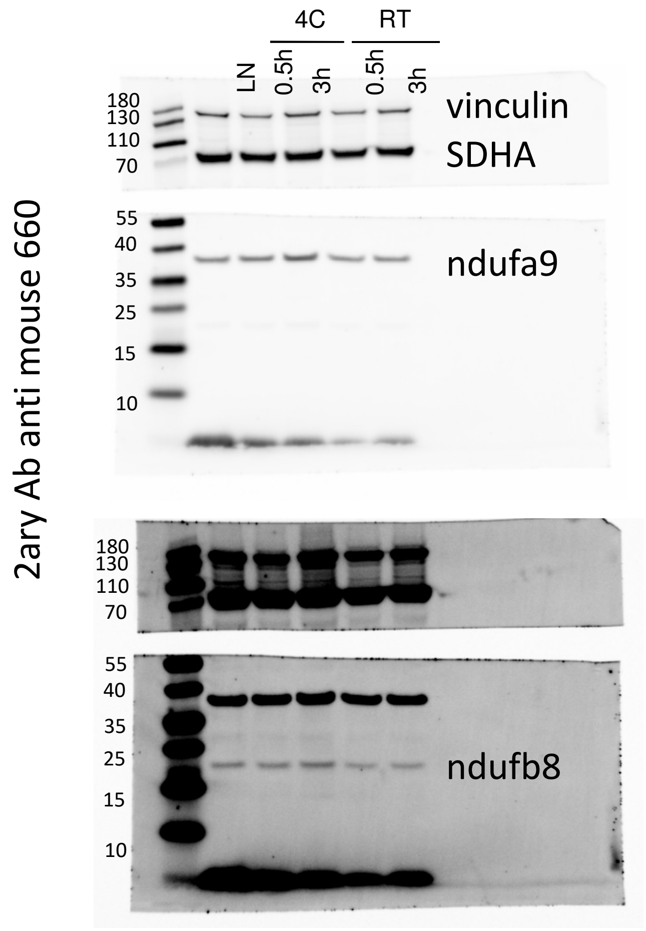

For ndufb8 , we used the same blot as above but enhancing the contrast

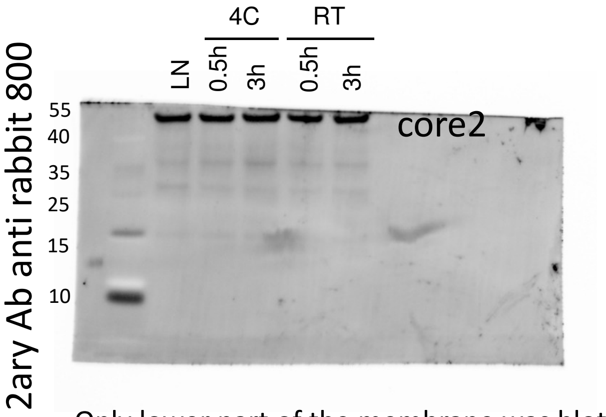

Only lower part of the membrane was blotted for core2
