# Supplementary figures and images for: A novel approach to measure mitochondrial respiration in frozen biological samples
Source: EMBO J. 2020 May 20;39(13):e104073. doi: 10.15252/embj.2019104073 (PMC7327496; doi:10.15252/embj.2019104073)

Supplementary Figure 5D

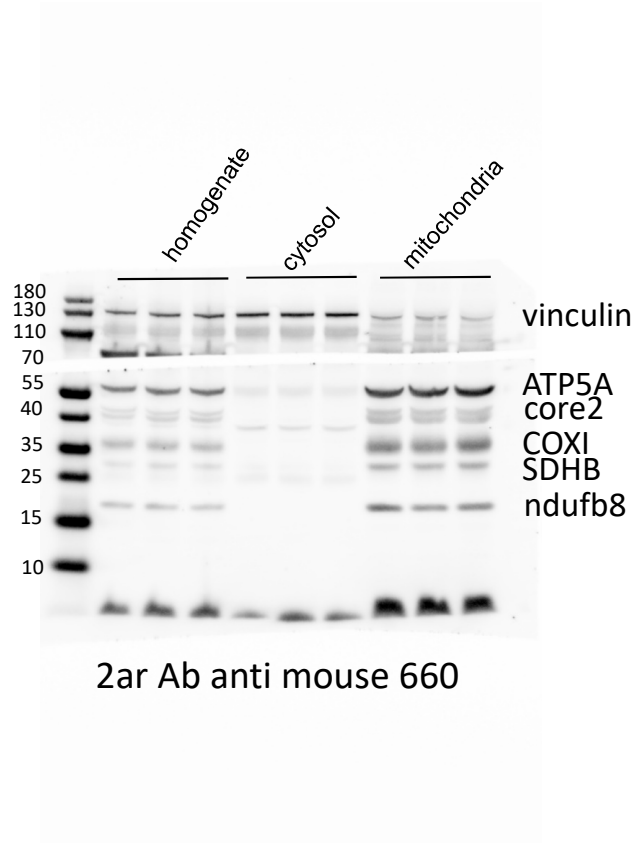

Supplement: Supplementary file 2 — Source Data for Appendix [file EMBJ-39-e104073-s006.zip › Appendix_SF5_blots.pdf]

Figure 4F: liver samples were used in Figure 4F, heart samples were used in Figure 7D

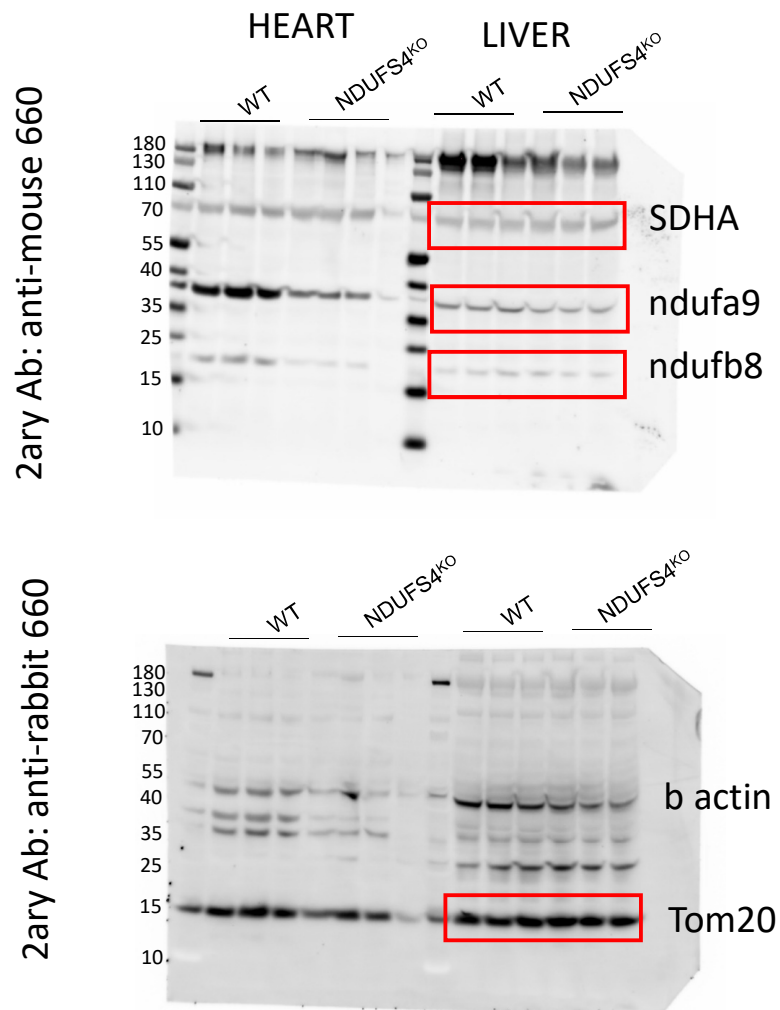

Supplement: Supplementary file 4 — Source Data for Figure 4 [file EMBJ-39-e104073-s002.pdf]

**Figure 5F**

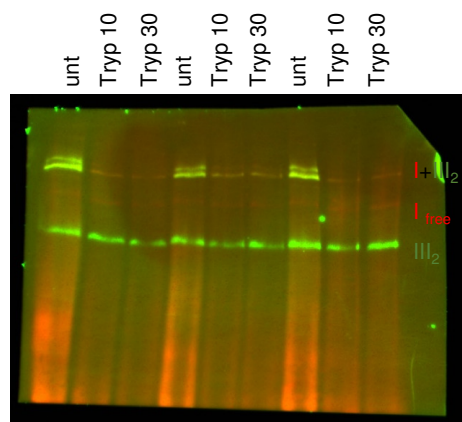

Core 2, **ndufa9**

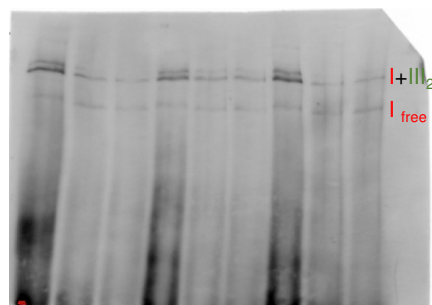

ndufa9

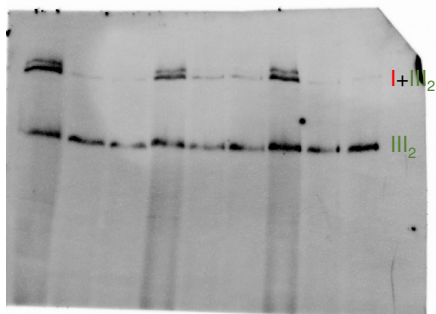

core2

**Figure 5G**

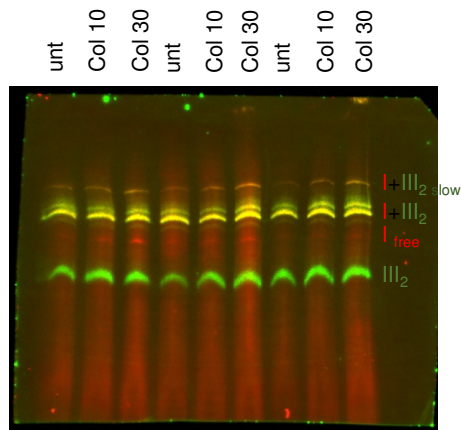

Core 2, **ndufa9**

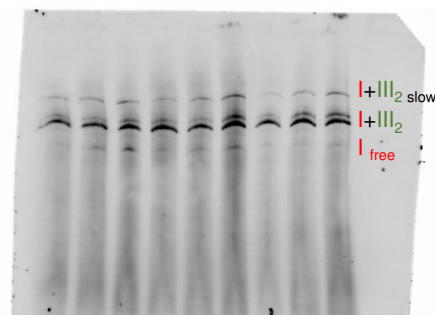

ndufa9

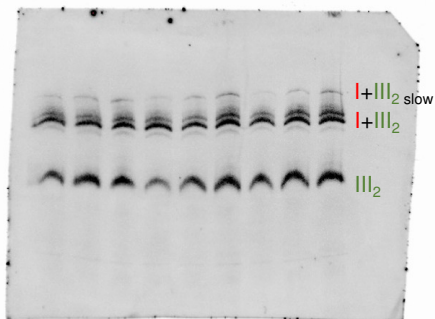

core2

**Figure 5H**

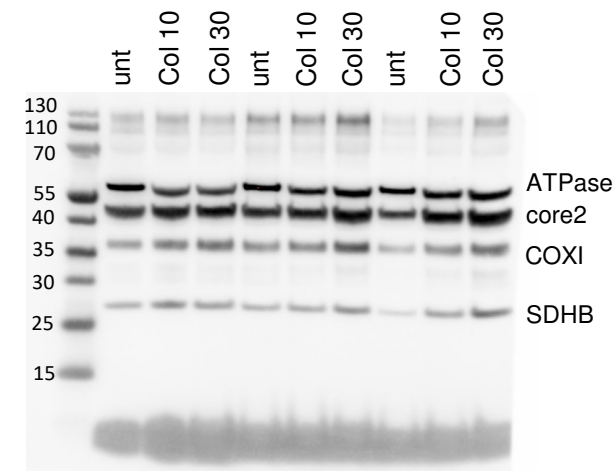

**Figure 5H**

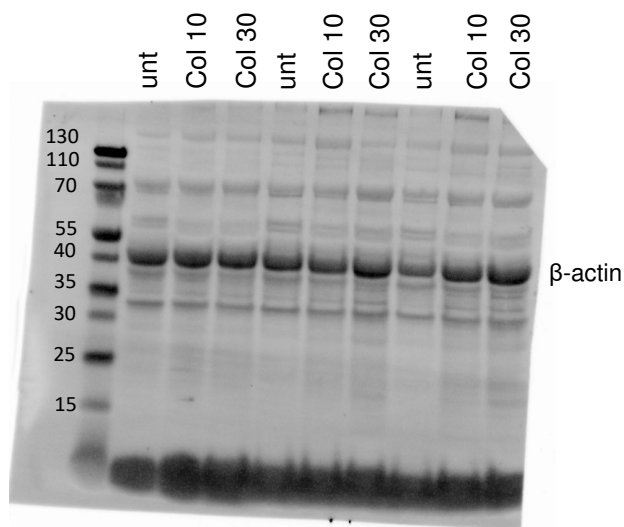

Supplement: Supplementary file 5 — Source Data for Figure 5 [file EMBJ-39-e104073-s003.pdf]

**Figure 6E**

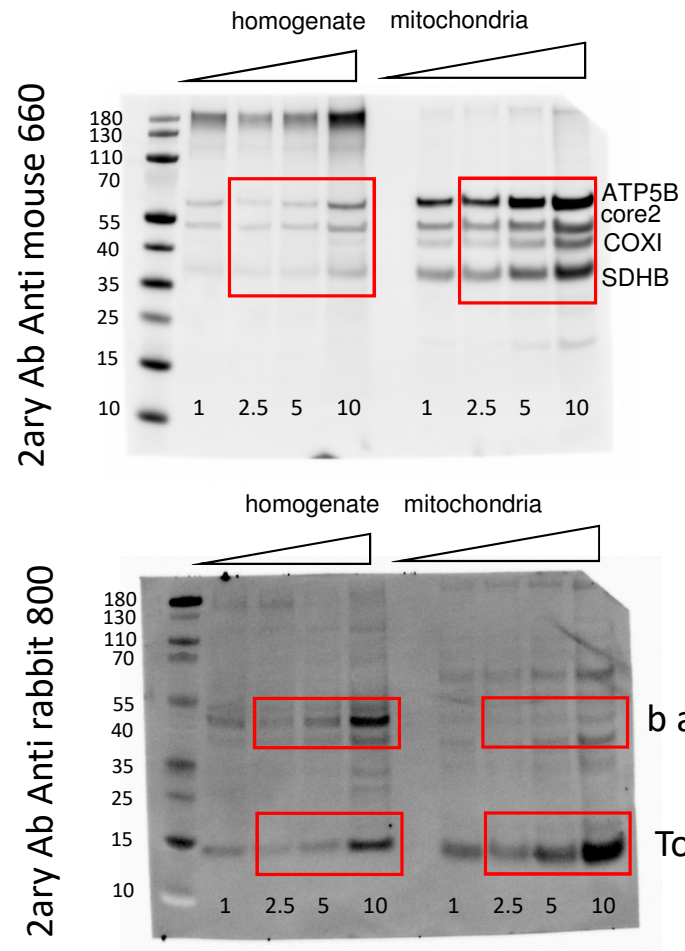

**Figure 6J**

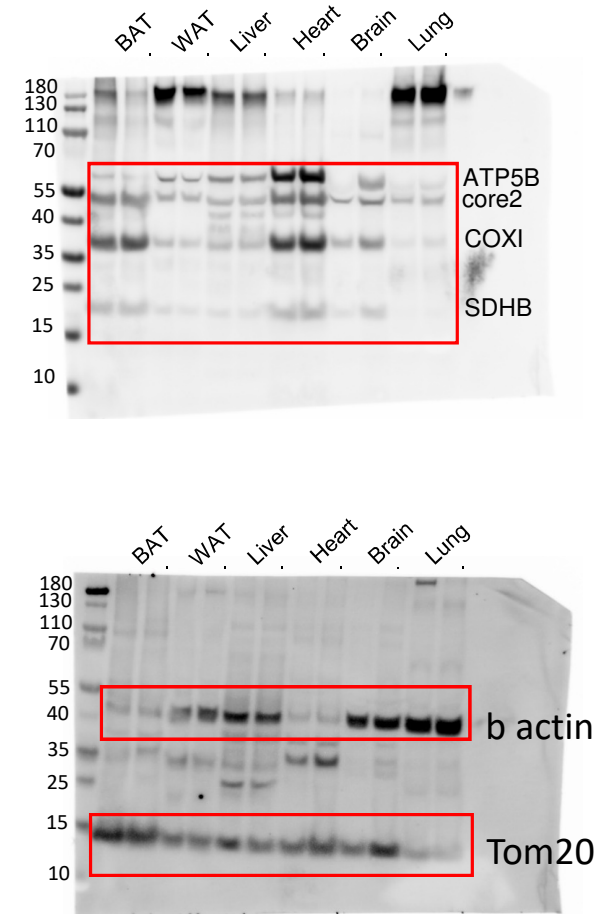

Only lanes 3, 4 5 for hom and 8, 9 10 for mitochondria were shown in the paper

Supplement: Supplementary file 6 — Source Data for Figure 6 [file EMBJ-39-e104073-s004.pdf]

**Figure 7D:** Heart lanes were used in Fig7D

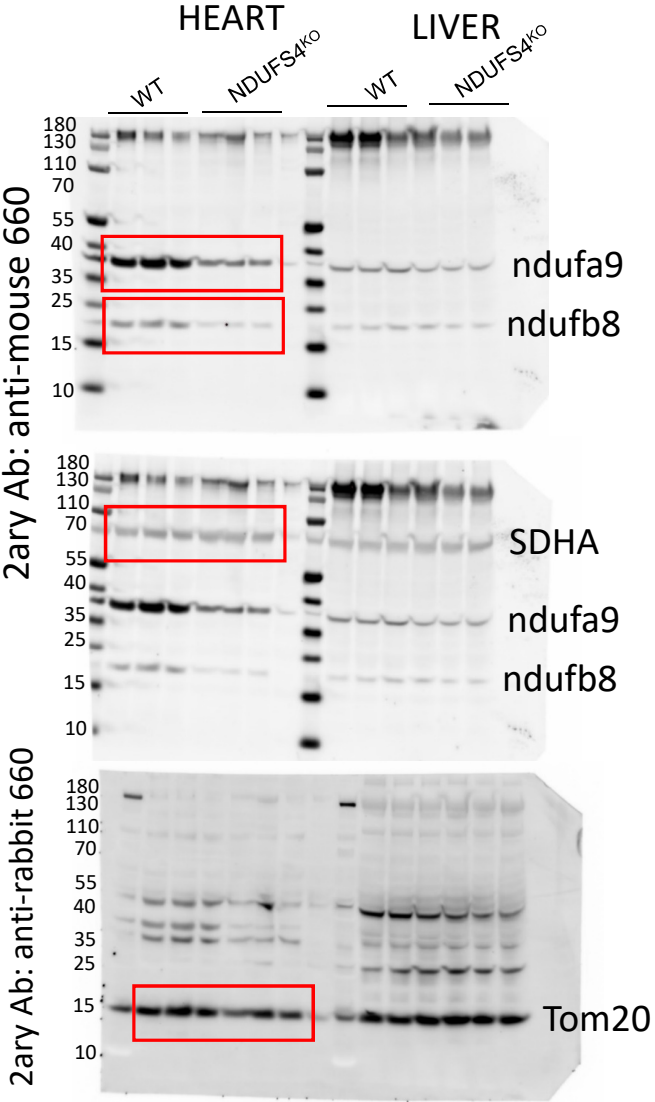

**Figure 7H**

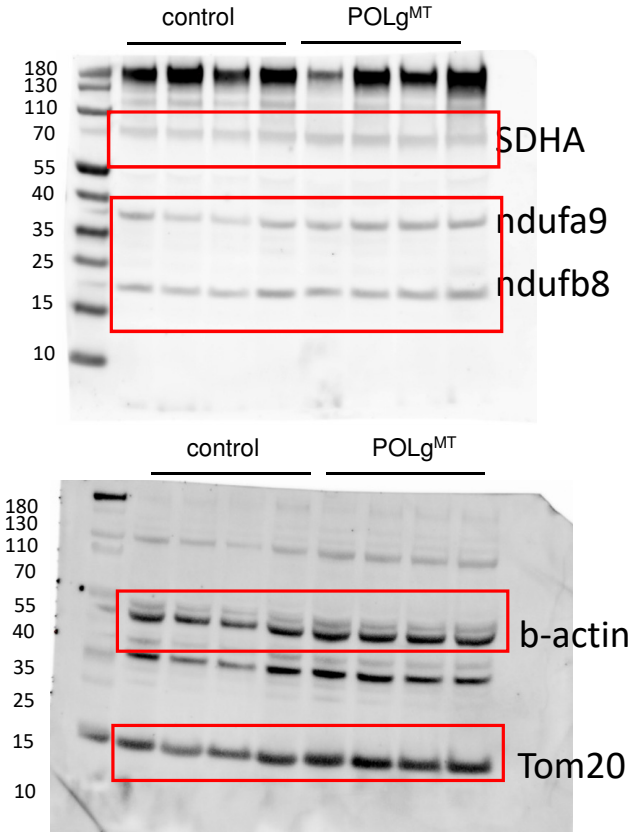

**Figure 7N**

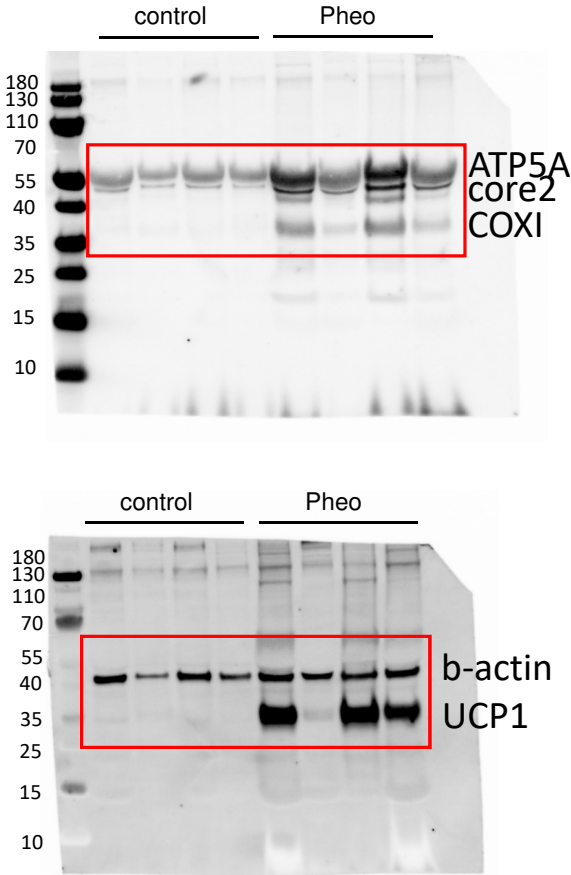

Supplement: Supplementary file 7 — Source Data for Figure 7 [file EMBJ-39-e104073-s005.pdf]
